# China catches up in commercial space: an interview with Ji Wu

**DOI:** 10.1093/nsr/nwac065

**Published:** 2022-04-13

**Authors:** Ling Xin

**Affiliations:** Ling Xin is a science writer based in Beijing

## Abstract

China continued to gather momentum in space in 2021. With a record-breaking 55 orbital launches, the country made it again to the top of the list, ahead of the USA’s 51 and Russia's 25 launches. However, nearly 90% of the Chinese launches were carried out by the state-owned Long March rocket family. In contrast, only about one-third of the US launches were made by government-funded rockets. *National Science Review* invited Ji Wu (吴季), an expert in space science, former director of the National Space Science Center (NSSC) of the Chinese Academy of Sciences and author of The Moon Summit, Lunar Hotel and Space Tourism, to talk about the internal and external driving forces behind the emerging private space sector in China, and government policies supporting its development since 2014. Wu also shared his insights into space tourism as a gateway to space for all, as well as his concerns for the orderly competition in the global market.

## THE NEW SPACE

**NSR**: Most space activities in human history have been initiated and funded by government. Can you walk us through how commercial space emerged?

**Wu**: Let's first take a quick look at how space activities began. More than a century ago, Russian rocket scientist Konstantin Tsiolkovsky proposed that the escape velocity from the Earth into orbit was about 8 kilometers per second. His idea was first put into practice during the Second World War, by a German engineer named Wernher von Braun who helped develop the V-2 ballistic missile. V-2 had a liquid-propellant rocket engine and was powerful enough to go above the atmosphere to hit London. When the war ended, von Braun moved to the USA and later became deeply involved in the space programs there. Actually, he led NASA’s development of the Saturn V rocket for the Apollo missions, landing multiple American astronauts on the moon for a decisive victory in the space race against the Soviet Union.

It is apparent that military needs and political will played a defining role in space activities from the start. In the 1970s, satellite communications found civilian uses and gradually separated from military-related space activities. Remember, there were no optical fibers back then, and people used communications satellites to make cross-continental telephone calls. Intergovernmental organizations such as INTELSAT (International Telecommunications Satellite Organization) were established to own and manage the satellites and provide broadcast services. The management of these services was commercialized, but the manufacturing of satellites and rockets remained very much a government business.

Soon after that, in the mid-1980s, private companies were allowed to provide launch services to the government in the USA. Around the year 2000, the market opened up and a swarm of entrepreneurs including Elon Musk entered the industry. Private spaceflight companies mushroomed. These companies focused on developing rockets and satellites, providing customized remote-sensing images and so on. Meanwhile, low-cost small satellites were being developed, first for educational purposes and then for commercialization. Every part of a CubeSat was turned into a standardized, off-the-shelf product. When students could buy parts and assemble a satellite on their own, satellite technologies came within reach.

**Figure 1:**
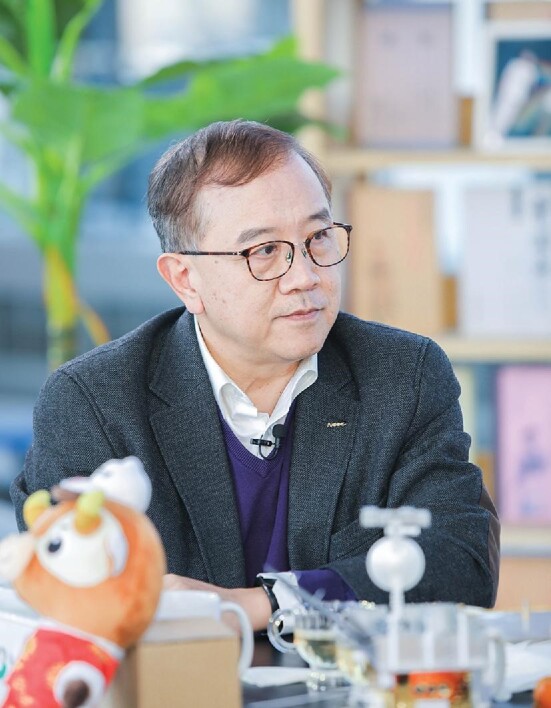
Ji Wu, former director of the NSSC, Chinese Academy of Sciences *(courtesy of NSSC)*.

When Elon Musk started out, he was looking to beat China's Long March rockets in terms of cost. He had planned to buy rocket engines from the Russians, but it didn’t work out. His team developed their own engines and went on to make reusable rockets. His company now charges $2000–3000 for one kilogram of payload launched into orbit. That's significantly cheaper than, say, the space shuttle, which costs $50 000 per kilogram, or the Long March series, which has remained at $16 000–32 000 per kilogram per launch over the years.

Private companies brought new approaches to the space industry. For instance, they abandoned the ‘no failure’ mindset of conventional, government-funded projects. If a government is going into space with tax payers’ money, they had better not blow up that money. Everything gets super complicated and the costs keep going up. And the more expensive a project gets, the more afraid people become of failure. This vicious cycle will be there as long as space is done with government money. In contrast, entrepreneurs use private investment, and their goal is to speed up research and development by learning from failures. Musk's company failed multiple times developing reusable rockets. This is a key difference between government-funded space projects and those done by the private sector.

**NSR:** How do you see the relationship between government-funded space and commercial space?

**Wu**: There are certain areas that are more suited to being taken care of by the government. I’m talking about space technologies related to public services, such as telecommunications, navigation and remote sensing. It's been the government's responsibility to provide disaster relief, build transportation infrastructures, survey agricultural resources and so on. Such services benefit all citizens and should be done with government money. For instance, weather forecasting is a typical public service. It would be hard to commercialize, and to make a profit from it. While there are customized needs such as accurate marine weather forecasts for sailors, the market share is quite limited.

The commercial space market has been slowly and cautiously opening up under government guidance. As private companies enter the landscape, they shake up the monopoly of state-owned enterprises and bring down the costs of going into space. However, if the market still relies heavily on government orders, it is hard for commercial space to see real, exponential growth. For instance, NASA’s annual budget is $20–25 billion. With companies like SpaceX, NASA can do a few more projects and do things more efficiently, but the cake is not much bigger, so to speak.

Therefore, private companies in the USA and Europe have been looking for their own markets and customers based on purely civilian needs. Some called it the search for ‘new space’. The new space is projected to be worth trillions of dollars, way larger than the old space market. Finding a pathway to the new space era has become the dream of every private spaceflight company.

**NSR:** Have companies had any luck with the search, or at least found some promising directions?

**Wu**: Right now there are two major directions to explore. One is the utilization of resources in space, or space mining. People thought about digging up precious metals from outer space and bringing them back for sale, but apparently that wouldn’t be very profitable. After all, it's hard to find an asteroid made of platinum or gold. Such materials form under very high temperatures

Imagine that we have all the necessary infrastructures built on the Moon, and rocket tickets are not terribly expensive any more—I’m sure some people would be eager to visit it.

—Ji Wu

and pressures, and usually exist in the interior of larger planets, not asteroids. Many space mining companies are now focusing on lunar resources. For instance, they are developing technologies to extract water from the surface of the Moon and turn it into fuels for rocket engines. It's actually easier and cheaper to lift off from the Moon than from the Earth because the Moon's gravity is about one-sixth of the Earth's.

The other direction is space tourism. Imagine that we have all the necessary infrastructures built on the Moon, and rocket tickets are not terribly expensive any more—I’m sure some people would be eager to visit it. What's really promising with space tourism is that it has a market that will not overlap with, and is in fact quite beyond, government responsibilities. The government takes care of public services and national security, but space tourism is all about tourism.

Why would an average person want to go into space? Tsiolkovsky said that the Earth is the cradle of humanity, but one cannot remain in the cradle forever. Space has simply taught us so much. When the first astronauts looked back on Earth from above, they saw no borders and they heard no arguing. It was simply a beautiful home planet for all human beings. This is a unique insight we get from space; most of us are unaware of that when bound to Earth.

The furthest that human beings have been in space is the Moon. Altogether, 24 people have reached the Moon; half of them set foot on the lunar surface. But the human species hasn’t traveled that far in five decades. The Apollo program was very successful, but it was more of a political project and subjected to political needs and funding. Today, private spaceflight has a real opportunity. The idea of space tourism is more than space companies making money and tourists taking pictures from space. It offers ordinary people the chance to see things in a very different light, like never before. In this sense, space tourism is meaningful for the advancement of human society.

## CHINA ON THE HORIZON

**NSR:** China is a latecomer to commercial spaceflight. What are the driving forces behind space companies in China?

**Wu**: China developed its first satellite in the 1960s, but systematic space activities only began in the 1990s. In recent years China has made good progress with manned spaceflight, lunar exploration, space science missions and other government-led programs.

The internal driving force for private space companies in China is an economic one: to reduce manufacturing costs and make profits. When I was leading the proposal and implementation of China's first space science missions in the 2000s, we had very few options when it came to rocket and satellite providers. Some state-owned enterprises even raised their bid every time we asked for a price. There were some microsatellite companies, but they all had government backgrounds.

In 2014, the State Council issued the so-called ‘Document 60’ (Guiding Opinions on Innovating Investment and Financing Mechanisms in Key Areas and Encouraging Social Investment,  关于创新重点领域投融资机制鼓励社会投资的指导意见). It allowed for more freedom in private investment in technology domains such as rocket launches and satellite manufacturing. Document 60 officially raised the curtain on commercial space in China. Since then, there have been over 200 registered companies in China, covering almost all aspects of the industry: launcher systems, rocket engines, satellites and subsystems, Telemetry, Tracking and Control (TT&C) and launch services, remote-sensing applications, navigation applications and so on. The annual venture investment has been standing at a few billion RMB during the past few years.

**NSR:** What are the external driving forces?

**Wu**: I think that's Elon Musk. His success with reusable rocket technologies really inspired the entire space industry, including companies in China. Even people at state-owned enterprises started to believe that private spaceflight is going to go somewhere, and some government-employed engineers quit their jobs to join the private sector.

But we need to see that the entrepreneurs in China are still exploring and accumulating experience. A lot of them are young and ambitious, and have worked in the IT or related industries, but need time to understand how the space industry works. They also need time to experiment with new technologies. For those who worked for state-owned enterprises, especially, they now have less restrictions and more opportunities to innovate. Let's give it 5 to 10 years for Chinese companies to mature and the market to straighten out.

**Figure 2:**
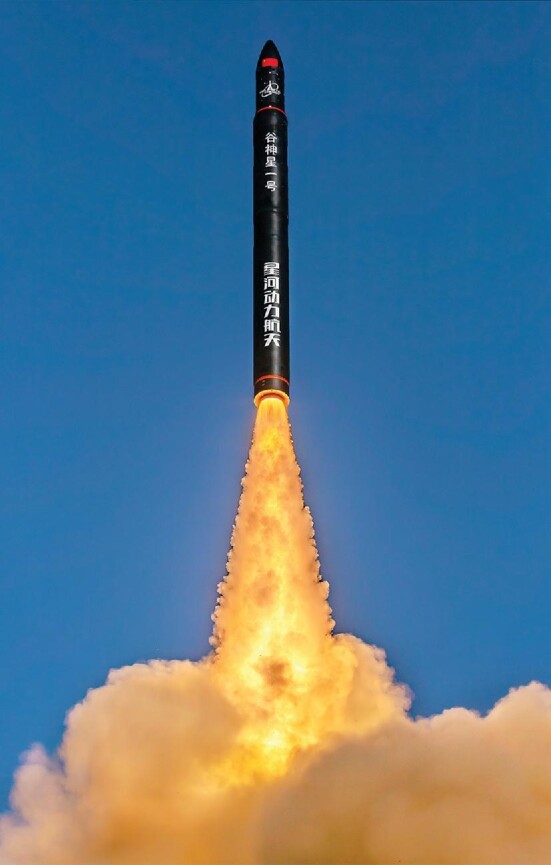
Ceres-1 (谷神星一号), a solid fuel orbital rocket designed by Chinese company Galactic Energy (星河动力), took its maiden flight from Jiuquan Satellite Launch Center in November 2020. (*Image: Galactic Energy*)

**NSR:** Besides Document 60, what other documents or policies did the Chinese government issue to encourage the private space sector?

**Wu**: There have been a dozen or so documents to boost or regulate private investment in space. For instance, the Medium- and Long-Term Development Plan for National Civilian Space Infrastructure (2015–2025) (国家民用空间基础设施中长期发展规划 [2015–2025]) laid out priorities for the civilian space industry. The Notice on Promoting the Orderly Launch of Commercial Vehicles (关于促进商业运载火箭规范有序发展的通知), issued in 2019, further regulated commercial space launches. Also in 2019, the Industry Catalogue Encouraging Foreign Investment (鼓励外商投资产业目录) opened up a number of previously closed industries to foreign investment, including satellite manufacturing.

Every five years since 2000, the State Council releases a white paper on China's space activities, in which we can see an increasing level of support from the government. The latest white paper was published in January 2022. It stated that the government is going to ‘expand the scope of government procurement of space products and services, grant relevant enterprises access and sharing rights to major scientific research facilities and equipment, and support these enterprises in joining the research and development of major engineering projects’. Citing the white paper at a press briefing, officials from the National Space Administration of China also mentioned that the country will cultivate space tourism, space biopharmaceuticals and new forms of space economy to promote the scale and efficiency of the space industry.

However, all these documents are guidelines and we do not have dedicated regulations for the commercial space sector yet. The government needs time to ponder over policy details to best encourage development and avoid unfair competition. As a member of the national committee of CPPCC (Chinese People's Political Consultative Conference), I have submitted a proposal to urge the formulation of related regulations as soon as possible.

**NSR:** What does commercial spaceflight mean to the development of space science in China?

**Wu**: The space science community in China embraces a fast-growing commercial space sector. If manufacturing and launch costs can come down, we will be able to launch more satellites and space telescopes, have more data to work with and make more research progress.

Space science helps with the development of private space too. For example, China is working on missions to the Moon's south pole. By comparing rocks from different regions on the lunar surface, scientists aim to understand the composition and distribution of natural resources there, and provide insights on how to best exploit them. The ongoing human health experiments on the International Space Station and the China Space Station can also provide valuable suggestions for space tourists.

## SPACE FOR ALL

**NSR:** I wonder what you think of SpaceX’s Starlink project, which has been criticized by astronomers.

**Wu**: I’m not happy about it, either. As I mentioned, telecommunications is a typical public service and government responsibility. That's because it's a dual-use technology: it can be used for civilian purposes and military applications. We don’t know if the company has fully considered all possible scenarios, but government and military involvement may be inevitable once the satellites become operational. The company probably had not considered how their satellite constellations would affect astronomy observations, either.

Beyond these concerns, Starlink is going to dramatically worsen the space junk problem. What will it be like to have 40 000 satellites packed in low Earth orbit? At this point we have a little over 6000 satellites in total, and we are already getting frequent collision warnings. Starlink will pose a big threat to space security. One collision can produce hundreds of pieces of debris, and some of the debris go on to collide with other objects. This cascading effect has been predicted to happen when the density of objects in low Earth orbit is high enough. What happens next? Low Earth orbit will be totally blocked, and no one will have access to space until the debris is absorbed by the atmosphere. This process takes several decades and will be a miserable disaster for all of us.

**NSR:** How long would it take for private companies to become profitable based on pure market needs?

**Wu**: Very soon, I believe, for leading space tourism companies in the USA and Europe. The market is already very hot, and rich people are lining up to pay millions of dollars for a ticket. That's good money to make in the first wave of space tourism. A bit later, these companies will probably lower the ticket price so more people can afford it.

**NSR:** Where will China's private space sector be 10 years from now?

**Wu**: It will take about five years for Chinese companies to catch up with reusable rocket technologies. I was told that several of them will roll out the first tests of reusable rocket engines or even the whole system soon. They are now making more effort than state-owned enterprises, since they are strongly motivated to reduce launch prices and stand out amongst fierce competition. As far as I know, many of them also aim to enter the space tourism business later. When the China Space Station retires in the early 2030s, there will be technology transfer to further boost space tourism in China.

**NSR:** How do you see the future of China's space activities in general?

**Wu**: When it comes to space, China has been basically working on its own and at its own pace. So far, so good. In the past decade China made the first landing on the far side of the Moon in human history, and brought back Moon rocks that are very different from the Apollo samples. China also landed on Mars at its very first attempt, a historically challenging task for any nation. Of course, these successes benefited from the scientific data collected by the previous space missions of the USA, Europe and so on.

So far, we are largely following other countries’ steps. Even when we land an astronaut on the Moon, that will be following others’ steps, not a first. Going forward, will China be able to make some firsts in deep space exploration? Let's wait and see. We can certainly expect robust government investment in a Moon base and manned missions. As for private space, the government is working on more specific, explicit policies to support its orderly growth.

